# Novel Drug and Gene Delivery System and Imaging Agent Based on Marine Diatom Biosilica Nanoparticles

**DOI:** 10.3390/md20080480

**Published:** 2022-07-27

**Authors:** Hanaa Ali Hussein, Muhammad Shahid Nazir, Nizakat Azra, Zeenat Qamar, Azman Seeni, Tengku Ahmad Damitri Al-Astani Tengku Din, Mohd Azmuddin Abdullah

**Affiliations:** 1 College of Dentistry, University of Basrah, Basrah 61004, Iraq; hanaazahraa85@gmail.com; 2 Department of Chemistry, COMSATS University Islamabad, Lahore Campus, Lahore 54000, Punjab, Pakistan; shahid.nazir@cuilahore.edu.pk (M.S.N.); azranizakat@gmail.com (N.A.); zeenatqamar90@gmail.com (Z.Q.); 3 Department of Toxicology, Advanced Medical and Dental Institute, Universiti Sains Malaysia, Bertam Campus, Kepala Batas 13050, Malaysia; azmanseeni@usm.my; 4 Department of Chemical Pathology, School of Medical Sciences, Universiti Sains Malaysia, Kubang Kerian 16150, Malaysia; 5 SIBCo Medical and Pharmaceuticals Sdn. Bhd., No. 2, Level 5, Jalan Tengku Ampuan Zabedah, D9/D, Seksyen 9, Shah Alam 40000, Malaysia

**Keywords:** biosilica nanoparticle, diatoms, drug delivery system, gene therapy, imaging agent

## Abstract

Mesoporous silica nanoparticles (MSNs) have great potential for applications as a drug delivery system (DDS) due to their unique properties such as large pore size, high surface area, biocompatibility, biodegradability, and stable aqueous dispersion. The MSN-mediated DDS can carry chemotherapeutic agents, optical sensors, photothermal agents, short interfering RNA (siRNA), and gene therapeutic agents. The MSN-assisted imaging techniques are applicable in cancer diagnosis. However, their synthesis via a chemical route requires toxic chemicals and is challenging, time-consuming, and energy-intensive, making the process expensive and non-viable. Fortunately, nature has provided a viable alternative material in the form of biosilica from marine resources. In this review, the applications of biosilica nanoparticles synthesized from marine diatoms in the field of drug delivery, biosensing, imaging agents, and regenerative medicine, are highlighted. Insights into the use of biosilica in the field of DDSs are elaborated, with a focus on different strategies to improve the physico-chemical properties with regards to drug loading and release efficiency, targeted delivery, and site-specific binding capacity by surface functionalization. The limitations, as well as the future scope to develop them as potential drug delivery vehicles and imaging agents, in the overall therapeutic management, are discussed.

## 1. Introduction

A DDS-based on nanoparticles (NPs) is essential in cancer treatment. Compared to conventional DDS, a NP-based DDS shows enhanced efficacy by increasing the half-life of the drugs or proteins for a slow release from the carrier, improving the hydrophobic drug’s solubility and allowing a controlled and targeted drug release at the diseased site [1]. Nanomaterials have found wide applications in synergistic platforms for cancer therapy such as photodynamic, immuno, chemo, and photothermal therapy, and for medical imaging, which includes computed tomography, magnetic resonance imaging, single-photon computed tomography, positron emission tomography, optical imaging, photoacoustic imaging, and ultrasound [2]. The major issues with the traditional pharmacological drug administration are a poor solubility, a short half-life, systemic toxicity, and drug disintegration before reaching the targeted site. The DDS therefore offers great potentials to overcome these constraints. Various types of nanocarriers have been developed for drug delivery as shown in Table 1, each with its own unique features, advantages, and disadvantages.

**Figure 1:**
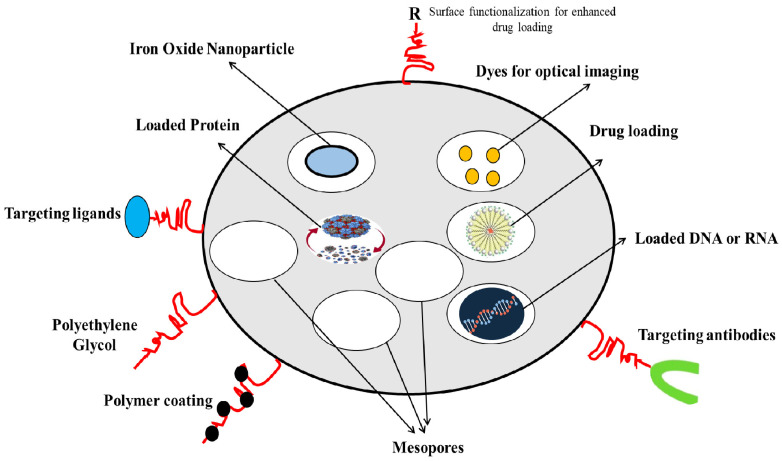
MSNs as versatile drug carriers (modified from [11] under Creative Commons Attribution (CC BY) license).

Silica and liposomes are the two most common materials used for targeted drug delivery. Mesoporous silica nanoparticles (MSNs) or silica nanoparticles (SiNP) have been applied for cancer treatment and diagnostic/imaging agents for cancer prognosis, such as the MSN-based biosensors and quantum dots, and for applications in tissue engineering, as catalysts in chemical synthesis, and as adsorbents for absorbing toxic substances and waste. Figure 1 depicts the versatility of MSNs as drug carriers. Silica is highly porous, has excellent drug-holding capacities, is biocompatible, and is prone to surface functionalization for tunable properties [3,4]. The large surface area and the tunable surface of MSNs can be loaded with drugs and multi-functionalized for targeting purposes, or carry traceable fluorescent or magnetically active agents for therapeutic delivery and medical imaging [5]. At the nano size, with a high pore size and surface area (often exceeding 1000 m^2^/g), the MSNs are superior for adsorption and drug loading [6]. The loading and release kinetics of the drug can be modified by changing the size of the NPs [7]. Surface modification of the MSNs could improve the targeting ability of the NPs, for more effective drug delivery, and for slow release to reduce systemic toxicity [8,9].

The synthetic methods of MSNs include the modified Stober method, fast self-assembly, dissolving-reconstruction, soft and hard molds, defrost and rebuild, and the modified aerogel process [10]. Numerous modifications have been made to produce nanoscale-ordered and nano-sized molecules. MSNs of various shapes and sizes can be synthesized in basic, acidic, and neutral conditions, by modifying the pH, surfactants, or polymers used, and the silica concentrations and sources [10,11]. As the biological membrane is the barrier to intracellular delivery, any efforts to deliver therapeutic/diagnostic agents to the diseased sites, must take cognizance of the assimilation mechanisms and transport of the carriers. The pathways for the penetration of the exogenous substances into the cellular membranes is possible via absorption, either through phagocytosis or endocytosis; and macropinocytosis [12]. Typically, the surface of the SiNPs is negatively charged due to the presence of a hydroxyl group after the hydrolysis of tetraethyl orthosilicate (TEOS). Amines, carboxylates, polyethylene glycol (PEG), and phosphate groups can be easily bound to the hydroxyl groups of the SiNPs by the hydrolysis of the corresponding silanes [13]. Surface modifications with different charges including PEG-SiNPs, OH-SiNPs, and COOH-SiNPs have been shown to influence the biotic distribution and secretion of SiNPs. The neutral-charged SiNPs (PEG-SiNPs), used to deliver drugs and imaging agents, show a relatively longer hemodynamic circulation and less uptake by the endothelial reticulocyte (RES) organs as compared to the other organs [14].

The major disadvantage of the chemical synthesis of SiNPs is the use of chemicals and the wastes generated thereafter. The chemical route is also time-consuming and energy intensive. Diatoms, an exclusive genus of algae, having sizes from 2 to 500 µm, are natural sources that can be used as alternatives to manufacture silica [15]. The shape of the diatom is just like a miniaturized pill box, where the cell wall is made of translucent, opaline silica, which can be absorbed from the surroundings and deposited as frustules in its biomineralized silica cell walls. Diatomite or diatomaceous earth (DE) is made up of the skeletal remains of the diatoms that have been fossilized [16]. Marine biosilica diatoms have a high aspect ratio, a high mechanical strength, and an excellent biocompatibility. Biosilica is low-cost, and diatoms can be grown in large-scale cultivation [17]. Marine biosilica has a sophisticated three-dimensional (3D) porous structure that is self-assembled and regularly structured, with multifunctional capabilities [18,19].

This review discusses the latest developments related to the use of the MSNs as nanocarriers in biomedical applications, with special focus on cancer therapy and diagnostics. The synthesis of these multifunctional NPs, surface functionalization, and the factors influencing the size and morphology, are elaborated.

**Table 1:** Applications, advantages, and disadvantages of various nanocarriers for drug delivery (modified from [20,21,22,23]).

Delivery System	Applications	Advantages	Disadvantages
Biopolymer-based nanoparticles.	Prostate cancer; improving skin elasticity; enhancing skin cell activation energy; tissue-engineering.	Prolonged drug delivery and circulation time in the body.	Mechanically weak; rapid degradability.
Solid Lipid-based Nanoparticles (SLNs).	Can be combined with different drugs; effective against different types of tumors (breast, lung, colon, liver, and brain).	Controlled release of drugs; increased bioavailability and biocompatibility of entrapped bioactive agents, good stability of unstable active components; enhanced skin hydration and penetration of drug.	Poor hydrophilic drug loading and releasing capacity; irregular gelation tendency, and high-water content.
Micelles.	Menopause hormone therapy; cancer therapy.	Sustained-release, less toxicity and can be removed by renal filtration; can draw the water-insoluble drugs into their hydrophobic core.	Instability in the blood stream; concentration reduced by blood dilution; the loaded drugs can leak out, reduced drug dosage at the targeted site.
Liposomes.	Anticancer activity; anti-fungal and protozoal infection; adjuvants in vaccination; signal enhancers/carriers in medical diagnostics and analytical biochemistry.	Decreased toxicity; increased delivery for smaller volume; biocompatible and biodegradable; ease of penetration in dermal layer.	High production cost; low stability and solubility; loss of drug.
Niosomes.	Pulmonary and protein delivery; cancer chemotherapy; carrier for hemoglobin; vaccine and antigen delivery.	Controlled and targeted drug delivery; osmotically active and stable; enhanced dermal penetration and oral bioavailability; nontoxic; biocompatible and biodegradable.	May show fusion, leakage, or hydrolysis of entrapped drug; low drug loading capacity; physically unstable; aggregation; expensive
Nanoemulsion.	Wastewater treatment; personalized medicine; 3D printing; biomedical and pharmaceutical applications	Higher stability and loading capacity; low production cost; suitable for hydrophobic drugs; control drug release.	Low permeability and bioavailability of drugs; low viscosity and spreadability.
Protein nanoparticles (Albumin, Glutamate, etc.).	Breast and pancreatic cancer; delivery of genetic materials, anticancer drugs, peptide hormones, growth factors, DNA, and RNA.	Enhanced solubility and delivery to tumor site; biodegradability; bioavailability; relatively low cost.	Large particle size; rapid degradation speed.
Dendrimers.	Antineoplastic; antibacterial; anti-inflammatory; cardiovascular therapy; imaging diagnostic.	Ease of functionalization; biocompatible; controllable molecular weight and size.	Low solubility but can be modulated by surface moieties.

## 2. Diatoms as the Natural Sources of Biosilica

Diatoms are single-celled phytoplankton that contribute to a fifth of all photosynthesis on the planet [15]. Diatoms can build micro- or nanoscale structures by depositing silica with distinctive architectures, a varied aspect ratio, and an ease of chemical functionalization/alteration to confer thermal and mechanical strength, optical/photonic properties, and environmental resilience [24]. These render the biosilica frustules with a wide potential for applications in bioimaging/biosensing, drug/gene therapy, photodynamic and fluid dynamics, biophotonics, and molecular filtrations. The non-porous biosilica generated from genetically modified diatoms has been developed for targeted drug delivery to neuroblastomas and B-lymphocytes, as well as a xenograft mouse model [25]. Mesoporous silica materials such as MCM-41 and SBA-15 have been widely exploited in the drug delivery applications [26], and the naturally engineered biosilica from diatoms offers a more cost-effective material for use as a drug delivery vehicle [27]. This has a superior biocompatibility, and a higher surface area, drug loading capacity, mechanical strength, and feasibility for functionalization in 3D structure [28,29].

For protection, diatoms have a mineral shell, and silica formed on the surface of the cell. Diatom morphology varies based on the species with a rod-like, hexagonal, or circular shape, and each species has its own distinct surface feature [27]. Among the diatom species are *Thalassiosira weissflogii*, *Thalassiosira pseudonana*, *Coscinodiscus* sp., *Nitzschia* sp., and *Aulacoseira* sp. [30]. The biosilica generation takes place in the “silica deposition vesicles” compartment. These vesicles consist of organic macromolecules that aid in the synthesis of silica and also serve as a template for frustule growth and the creation of incisions and dumps [31]. Biosilica frustules can be specific to the diatom species and the environment in which they are developed [32]. The silica morphogenesis can be altered such as by changing the processing of silaffins in *Thalassiosira pseudonana* [33]. A diatom frustule has a distinct nanostructure with diverse features such as spikes, holes, crests, networks, and bristles [34]. Diatoms can be divided into centric and pennate structures. Because of their extensive pore design and distinct porosity, the centric diatoms have a centrifugally proportioned structure. In contrast, the pennate diatoms have a more extended structure. The epitheca is the superior portion of the diatoms, while the hypotheca is the inferior portion [35].

**Figure 2:**
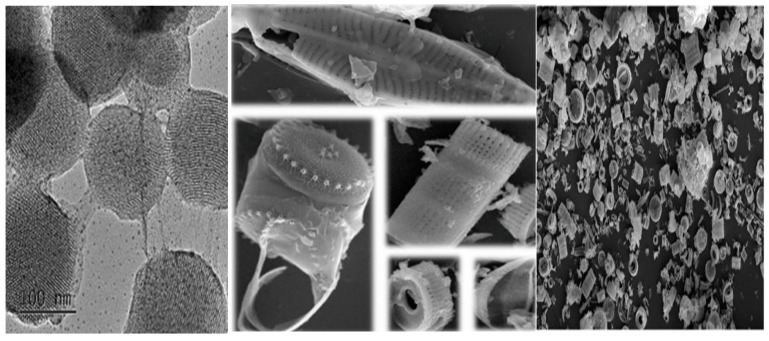
Diatom structure in the form of microcapsules as observed under light microscope; and the MSNs under transmission electron microscope exhibiting ordered porous structure (modified from [3] under Creative Commons Attribution (CC BY) license; and [15] with permission, Copyright © 2021, Elsevier).

The cultivation conditions of diatoms require the temperature of 20–24 °C, 18 h lights (1000–1200 lux) and 6 h dark, pH 8.2–8.7, and salinity 20–24 g/L, with culture mixing using an aerator [36]. In the natural environment, the microparticles of diatoms may include impurities such as clay, volcanic gas, terrigenous particles, and miscellaneous organic substances. These necessitate the removal of the contaminants from marine biosilica before pure silica micro-shells, as shown in Figure 2, can be recovered [37]. The amorphous structure [38], and the nano-pores encased in an ultra-fine polymer layer, render unique characteristics to become an inexpensive carrier of drugs and therapeutics [39]. Pure diatoms can be obtained by utilizing a pulverizer to crush the structure, with a compatible solution to remove the contaminants [30]. For surface modification, the organic layers can be removed, and the hydroxyl groups added on the surface [40].

## 3. Characteristics, Biodistribution, and Synthesis of MSNs

### 3.1. Properties of MSNs

Silica is “Generally Recognized As Safe” (GRAS) by the United States Food and Drug Administration (USFDA, Silver Spring, MA, USA) [3]. The characteristics of the NPs, such as shape, size, and porosity, play an important role in delivering drugs to the target site, in effective protection of the cargoes such as imaging agents, drugs, oligonucleotides, and enzymes, from premature release, and unwanted degradation in harsh environments, such as the stomach and intestines, before reaching the specified target and in subsequent removal from the body [3]. Size is the key factor controlling cellular uptake and biocompatibility of the NPs [41]. Three different forms of the MSNs, such as spherical nanoparticles (NS), long-rod nanoparticles (NLR), and short nanoparticles (NSR), have been analyzed for in vivo oral bioavailability where the NLR shows a longer residence time, a longer blood circulation, and a slower renal clearance as compared to the NS and NSR [42]. The NSR degrades faster as compared to the NS and NLR due to the higher specific surface area [43]. These ordered porous structures allow the precise control of drug loading and release kinetics (Figure 2) [3]. The large surface area (>700 m^2^/g) and pore volume (>1 cm^3^/g) give ample room for particle loading and improved drug solubility. The molecular size of the MSNs from 50 to 300 nm is suitable for easy cellular endocytosis by the living cells [3]. There are two functional surfaces—the cylindrical pore and the exterior particle surface—containing silanol that can be selectively functionalized or engineered with targeting bonds. The tunable diffusion and adjustable drug release make it feasible to deliver effective cell-specific drugs and for the development of a biogenic local concentration in the target area, thus reducing the total dose and preventing severe or chronic complications [3].

### 3.2. Uptake and Biodistribution

With intravenous delivery, the MSNs are normally distributed in the liver and spleen, and to a lesser extent in the lungs, and a small amount in the kidneys and heart [14]. Effective absorption of the MSNs involves energy-dependent cellular uptake and delivery [44], which can be controlled by the size and morphology of the NPs, the nano-carrier surface functionalization, and the electrostatic interactions between the MSNs and the cell membrane. The most suitable pathway to assimilate the MSNs is through cellular endocytosis, incorporating adherent material into vesicles (endosomes) [45]. For effective therapeutic activity, the MSNs should release the cargo in the cytoplasm via various endosomal escape routes [46]. Particles larger than 1 μm can cause phagocytosis that engulfs the particles into specific cells such as monocytes, neutrophils, macrophages, and dendritic cells. The particle size, shape, and surface charge affect one of the specific endocytic pathways [12]. The smaller particles (<200–300 nm) are absorbed by the endocytosis pathway, in most cases involving mechanisms such as clathrin- and cavulin-dependent cell endocytosis and caffeolin-dependent, based on the types of cells and surrounding conditions. Although the smaller particles may exhibit a longer circulation time, the blood circulation period, in vivo distribution, and biological filtration will further influence the impact of the particle size. The effects of the pore size, shape, and surface properties of SiNPs on the cytotoxicity against macrophages (RAW 264.7) and epithelial carcinoma (A549) cells have been evaluated [12]. The main pathway of the toxicity associated with SiNPs is attributed to its surface silanol groups, which interact with the membrane components, leading to cell lysis and the leakage of cellular components. The pyrolyzed MSNs for example, show a lesser interaction than the non-porous silica due to the lower density of the silanol groups on the surface. Three different pore sizes, 2.3, 5.4, and 8.2 nm, named MSN2, MSN5, and MSN8, respectively, have been prepared using microemulsion to evaluate the effect of doxorubicin (DOX)-loaded MSN pore sizes on the anticancer efficacy. The MSN2 exhibits the lowest loading capacity (8.2%), while MSN5 shows the strongest cellular uptake and release profile, suggesting the importance of controlling the pore size of the nanocarrier [47]. The NPs synthesized with a high surface area and a small size can improve the biodistribution of cancer drugs and increase the diffusion time in the bloodstream. The drug-loaded NPs can be designed to target and select only tumor cells, and show high permeability and retention prospects in the specific tumor environment [4].

### 3.3. Chemical and Biosynthesis

The MSNs can be synthesized using Stober’s, microemulsion, solution-based, and evaporation-induced self-assembly (EISA) methods. The materials of a mesoporous nature have been synthesized by changing the starting precursors and reaction conditions to result in materials with a varied structural arrangement or pore size. For instance, the Mobil Crystalline Material No.48 (MCM-48) has a cube arrangement, while the MCM-50 has a plate-like arrangement. Tri-block non-ionic copolymers such as poly(ethylene oxide) alkyl (PEO) surfactant and poly(alkylene oxide) block copolymers are also used as template designated as SBA-11 (cubic), SBA-12 (3-d hexagonal), SBA-15 (hexagonal), and SBA-16 (cage-shaped cube) based on the consistency of the mesoporous structure and the tri-block polymers used [48]. The SBA-15 differs from MCM in that it has pores larger than 4.6–30 nm with thicker silica walls [49] suitable for biomedical applications. FSM-16 is a mesoporous structure, prepared from quaternary ammonium surfactant as a template and polysilicate kanemate layers, suitable for pharmaceutical applications rather than as an adsorbent or for catalysis [11]. The time-consuming and energy-intensive processes and the wastes generated are among the major problems that hinder the further development of the chemical synthesis of the MSNs.

Nature has provided promising alternative porous materials from the biosynthesis of the MSNs. During biomineralization, marine organisms make remarkable, well-designed inorganic frameworks and patterns down to the nanometer scale [50]. Silica has important biological functions in higher plants, animals, and humans. The presence of silica in most cellular organisms such as bacteria and fungi proves its essential roles. Silica is actively taken up and transported by diatoms and algae for their replication and survival. The lack of silica in animals may result in the abnormal growth of tissues, especially collagenous tissues such as the skull, hair, peripheral bones, skin, and joints. Hence, the importance of biosilica in medical fields such as in orthopedics [51]. In fact, the compositional control of shape, size, pore pattern, surface area, and pore size of the synthetic MSNs, can be achieved in diatoms by altering the physicochemical parameters, post-harvest modifications, or genetic engineering [50]. Diatoms are considered to be the main biofactories for global silica biogenesis. The application of frustules has led to the creation of a new, rapidly developing field called “Diatom nanotechnology” [50]. The solid surfaces of frustules consist of particles with a size of approximately 40 nm or 100–200 nm and display a patterned network of highly ordered pores mostly of uniform shape (nano to micrometer in size) with a homogeneous distribution within the surface. These pores contain species-specific patterns, with unique molecular transport properties and mechanical, photonic, and optical properties [52,53].

Bacterial silica production is achieved by the interaction of soluble anionic silicates with amine side chains (the positively charged groups on the peptidoglycan layer). The bacterial membrane demonstrates the deposition of silicates as thin unorganized scales (100 nm) and as a granular covering on the cell wall. The interaction in the peptidoglycan may be based on the hydrogen bond between the polysaccharide hydroxyl groups and silanol groups. The interaction between mostly negatively charged bacterial cell walls and the external cations may provide nucleation sites for mineralization. This is often found in those bacteria living in extreme environments, suggesting their protective properties [52]. Marine sponges form specific types of amorphous silica structures known as spicules (ranging from microscopic to macroscopic structures). These spicules are formed in a separate cell type called sclerotic cells, with the help of proteins (scaffolding silicatein) that catalyze hydrolysis and the polymerization of tetraethoxysilane [54]. Other alternative methods, in addition to chemical- and biosynthesis for the fabrication of MSNs, may involve spray drying [55], pressured carbonation [56], and low-temperature vapor-phase hydrolysis [57], which also involve additional chemicals and energy input.

## 4. Surface Modification, Functionalization, and Engineering of MSNs

As drugs differ in their chemical and physical properties, it is difficult to load drugs into only one carrier and release them one by one in the body. It is crucial to be able to design a smart-rate and section-controlled multi-drug delivery system to enhance targeted delivery to the diseased sites [58,59]. For this, biosilica modified with different surface and coating materials could provide solutions [60,61]. Surface modification is one of the approaches to fine-tune the pore size and improve the bioavailability and drug control release from a mesoporous silica material [53]. Diatoms can provide solutions to overcome problems associated with water-insoluble drugs and poor bioavailability. The coating of biosilica is done layer by layer using polyacrylic acid or micro-particles of polyallylamine hydrochloride [62,63]. The introduction of cross-linkers to the surface of biosilica creates strong covalent interactions with proteins or nucleic acids, which is critical for some specific applications. Biosilica is resilient and stable, and therefore can be standardized to study drug release kinetics and to meet high surface-to-volume ratio requirements for drug delivery [64,65]. Although the surface area of diatom biosilica varies between 1.4 and 51 m^2^g^−1^, effective drug release is further influenced by the size, layout, and distribution of the pores [66]. The mechanisms may involve a fast release phase from the detachment of the weakly attached drug molecules to the biosilica surface, and a gradual release phase with drug transport from the carrier interior structure to the external environment.

Marine biosilica can be chemically modified to improve the delivery of soluble and non-soluble drugs [53]. The versatility can be improved by incorporating different ligands for tunable properties in biomolecular and cell sensing, intracellular delivery, and disease diagnosis. The high concentration of surface silanol groups of the MSNs can be modified with diverse organic functional groups. There are three well-defined domains that can be functionalized independently—the silica framework, the mesopores, and the outer surface of the NPs. These allow for nanoscale platforms to integrate multiple functionalities in treating and diagnosing diseases [67]. The functional groups could control the surface charge, chemically bind the functional particles inside or outside the pore, and control the pore size to trap the particles within the nanopores [68]. Some drugs are hydrophobic, which prevent them from penetrating the hydrophilic silica. Functionalization with hydrophobic groups could therefore enhance drug loading and prolong the kinetics of drug release from porous channels into the aqueous medium due to the low wettability of the material surface [69]. Both the internal and external surface of MSNs can be modified with different moieties, affecting the drug loading and release behavior, the tumor-targeting ability, and the imaging effect [69]. Some drugs may be limited to specific channels. In this case, higher loads and slower release kinetics can be achieved if the silica wall is functionalized with specific functional groups to cater for specific drugs, or a specific ligand to target a specific tumor. For instance, folate is affixed to the surface of the MSNs to target cancer cells with increased expression of the folate receptor [69].

**Figure 3:**
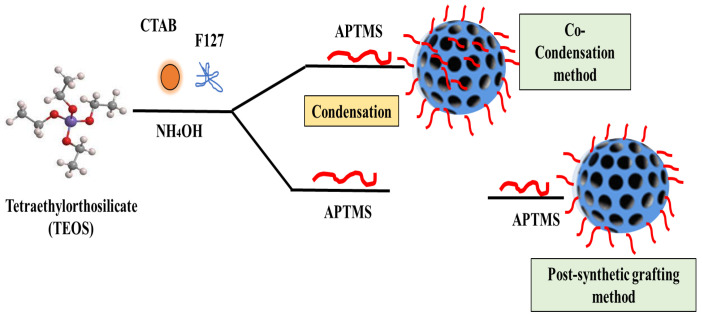
Surface functionalization methods of the MSNs based on surfactant displacement, co-condensation, and post-synthesis grafting methods (modified from [41] under Creative Commons Attribution (CC BY) license).

Surface functionalization of the MSNs can be attained by surfactant displacement, co-condensation, and post-synthesis grafting methods [41,68] (Figure 3). The co-condensation method entails the presence of modified functional groups even within the pores of the NPs, while post-synthetic grafting involves the coupling of functional groups, often on the surface of the NPs [41]. As shown in Table 2, the advantages of the co-condensation method include a simple process, a standardization in uniformity distribution, and a high achievable load. However, under some conditions, the recovery of the surfactants may not be complete, depending on the solvent used. The degradability and elimination of SiNPs can be carefully designed by changing the size, structure, and functional properties [41]. Factors that may affect the efficiency of the hydrolysis rates of SiNPs include the effect of surface modification, pH, pore size, and structure composition. The molecules with co-condensing functional groups (synthesized in the base state) could degrade at a much faster rate than the purely silicified MSNs. Additionally, when the co-condensation molecules are modified with disulfide-bridged silane on the surface, the degradation rate becomes slower [70].

During the post-synthesis grafting method, the functional groups are introduced after demolding by extraction or calcination [71]. This provides many possibilities for the functional group sites to be grafted with the most chemically sensitive organic functions and can be subjected to hydrolysis and removal reactions [72]. However, the distribution of the functional groups may not be regular if blocking of the nanopore occurs [68]. One of the major issues with the post-synthesis method is the potential for the mesopore openings being blocked by the functional groups, leading to a heterogeneous mesopore matrix [73]. A bi-functional surface modification of the MSNs, by incorporating the co-condensation and post-synthesis grafting method, may provide an alternative route of functionalization [74]. The coating method may have specific control of the stereochemical arrangement of the ligands on the mesopore surfaces [75].

The surfactant displacement method forms a single-layer uniform coverage with finely controlled amounts of functional organic silanes on the surface [68]. It consists of direct surfactant decomposition with simultaneous removal of the surfactant using acidic alcohol as a solvent [76]. This method synthesizes a homogeneous monolayer coating in which the number of functional groups can be precisely adjusted on the mesopore surface [67]. Lactose targeting is combined with drug delivery and cellular endocytosis of the MSNs to form a new DDS. Docetaxel as the model drug and fluorescein isothiocyante as the dye confirm that the cargo-loaded MSNs functionalization with lactose (hMSNs) attains a higher drug release over a long period of time, as compared to the cargo-loaded MSNs without surface functionalization [77].

**Table 2:** Description, advantages, and disadvantages of surface functionalization methods (modified from [74,75]).

Method	Description	Advantages	Disadvantages
Co-condensation (one-pot synthesis).	Co-condensation (precipitation) method uses various (organo) siloxane precursors in a one-pot (hydrothermal) sol–gel process.	Appropriate to a wide variety of organoalkoxysilanes;	Produced materials with less ordered structure.
		Suitable for various reaction conditions;	
	Allows preparation of organic–inorganic hybrid materials in a short time.	The coverage of functional groups is homogeneous;	
		High loading of drugs.	
Grafting (post-synthesis modification).	Carried out by silylation on free (≡ Si–OH) and geminal silanol (¼ Si(OH)_2_) groups.	Maintain a large amount of surface silanol groups after removal of the surfactant;	Non-uniformed distribution of the grafted groups on the surface.
		Higher concentration of functional groups in the final product.	
Imprint coating method.	Specific control of the stereochemical arrangement of the ligands on the mesopore surfaces;	Uniform distribution of pore size allows the formation of uniform imprints;	High amount of coating agent;
	Optimizes the binding of targeted metal ion.	Limits the number of other complexes.	Thickness of coating layer not easy to control.

Mesoporous NPs at different sizes, volumes, and porosities exhibit different levels of toxicity at single-dose, acute (10 days), and near-chronic (60 and 180 days) dose exposure to mice [78,79]. The smaller non-porous SiNPs (50 nm) and larger mesoporous SiNPs (500 nm) exhibit more severe toxicity than the larger non-porous SiNPs (500 nm) [79]. Through surface engineering or the surface coating of MSNs such as with hyaluronic acid (HA), targeted drug deliveries to cancer stem cells can be attained [70]. Surface coating by positively charged polymers such as polyethylenimine (PEI) could attract negatively charged nucleic acids. The nucleic acid binding can be so strong that it can protect from nuclease activities and this can be useful for the delivery of plasmid DNA or short interfering RNA (siRNA) [69]. MSNs have been used to stabilize lipases, which are the common enzymes for the hydrolysis of triacylglycerol to fatty acids. The MSNs confer the combination of mechanical and thermal stability with controlled compositional properties and abundant surface hydroxyl groups that allow surface engineering for the strong immobilization of lipases with hydrophobic supports [80]. A silica aerogel (SA–OH) nanostructure has been modified by grafting the –COOH group into SA–COOH. The loading amount of celecoxib (CCB) drug is increased with SA–COOH, where the bonding between COO– on the silica with NH_3_ of the drug is improved. Both drug-loaded SA–OH and SA–COOH exhibit greater loading capacities and faster drug dissolutions as compared to the pure drug. The controlled release rate is observed in the case of SA–COOH/CCB as compared to the SA–OH/CCB, suggesting a good potential for tunable drug dissolution and biocompatibility [81].

**Figure 4:**
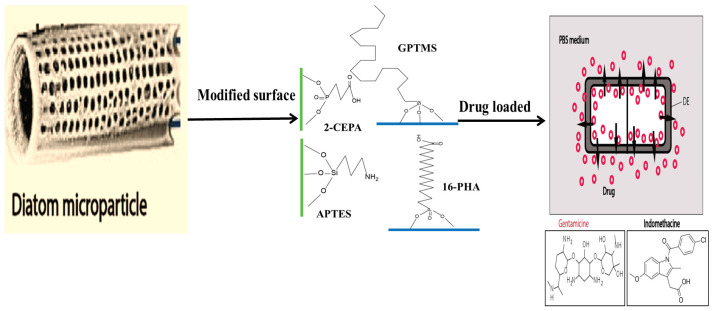
Schematic illustration of diatom microparticles with different organosilane functionalizations (APTES, GPTMS) and phosphonic acid (2-CEPA, 16-PHA) to increase the hydrophilicity or hydrophobicity of surface for the release of indomethacin (hydrophobic) and gentamicin (hydrophilic) drug from diatomaceous structure [26] (modified with permission, Copyright © 2014, Elsevier).

**Figure 5:**
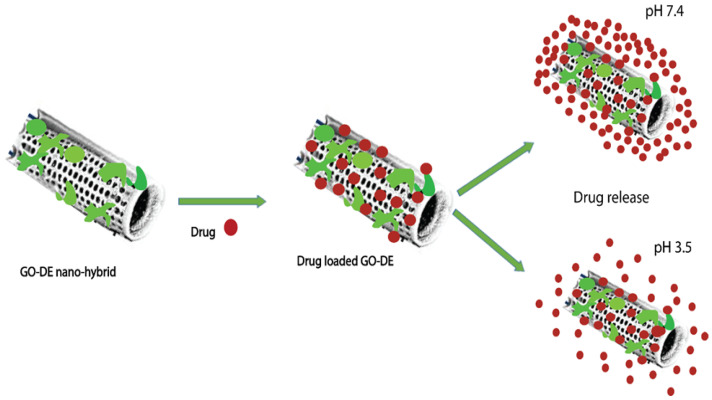
Loading of IMC onto GO–DE nanohybrid depending on the pH of the medium [83] (modified with permission, Copyright © 2013, Royal Society of Chemistry).

The binding of different antibodies to the surface-engineered diatom silica from *Coscinodiscus wailesii* has been demonstrated where the biological functions of the antibodies are retained. The modified silica exhibits prospective applications in antibody engineering similar to immunoprecipitation [82]. As shown in Figure 4, the drug loading and release capacity can be changed from surface-engineered diatomaceous earth (DE) microparticles [26]. Different types of silanes such as 3-aminopropyl triethoxysilane (APTES), methoxy-poly(ethyleneglycol) silane (mPEG-silane), 7-octadecyl trichlorosilane (OTS), 3-glycidyloxypropyl trimethoxysilane (GPTMS), and two phosphonic acids, namely 2-carboxyethylphosphonic acid (2 CEPA) and 16-phosphonohexadecanoic (16 PHA), are attached to the DE surface. The hydrophilic surfaces favour the extended release of the water-insoluble drug indomethacin (IMC) while the hydrophobic surfaces favor the sustained release of water-soluble gentamicin [26]. Novel nanohybrid particles from DE and graphene oxide (GO) have been synthesized where the GO is attached to the DE using covalent coupling and electrostatic attraction. The hybrid material is used as a smart pH-dependent drug microcarrier at pH 7.4 and 3.5, using IMC as the model drug (Figure 5) [83].

Biosilica from *Thalassiosira weissflogii* is functionalized with a good reactive oxygen species (ROS) scavenger, 2,2,6,6-tetramethylpiperidine-*N*-oxyl (TEMPO), and then explored as a carrier for ciprofloxacin, an orthopaedic and dental drug. The antioxidant property of ciprofloxacin is enhanced by silica functionalization with TEMPO [30]. Diatomite nanoparticles between 100 and 300 nm are surface engineered with 3-aminopropyltriethoxysilane and labeled with tetramethylrhodamine isothiocyanate. The confocal imaging analyses suggest that the distribution of nanoparticles is homogenous in the cytoplasm and the nucleus, which is ideal for DDS [84]. The diatom microcapsules with different hydrophilic and hydrophobic functionalizations have resulted in an increased water-insoluble IMC drug loading capacity by 60% with *N*-[3-(trimethoxysilyl)propyl] ethylenediamine, where the interaction of active polar functional groups on the silica enhance the drug loading capacity and prolong drug release [85]. Natural, mesoporous, and biodegradable silica from diatom *Amphora subtropica* (AMPS) has been extracted and purified as *Amphora* frustules (AF), which are then surface engineered with chitosan and tethered with DOX. The steps involved the demineralization of AMPS (AF) to form Amino-AF (AF-NH_2_), where APTES and glutaraldehyde are used as cross linkers to bind AF and Chitosan (Chi) to synthesize Chi@AF. The result suggests an efficient drug loading and release and a high biocompatibility and is therefore a good candidate for DDS [86].

## 5. Cytotoxicity and Anticancer Activities of Drug-Loaded MSNs

**Figure 6:**
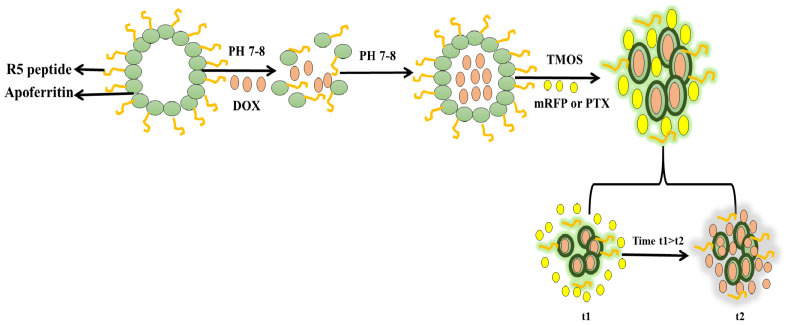
Schematic diagram of compartment-based and rate-controlled dDDS with PTX-DOX drug combination [91] (modified with permission, Copyright © 2020, Elsevier).

The synthesis of diatom nanoparticles (DNPs) with a functionalized nanocarrier can replace synthetic NPs for their application as an anticancer DDS. Three different surfactant- templated biosilica NPs have been synthesized—Triton X-100 (Triton@MSNs, nonionic surfactant), sodium dodecylbenzene sulfate (SDBS@MSNs, anionic surfactant), and cetyltrimethylammonium bromide (CTAB@MSNs, cationic surfactant)—as carriers of an anticancer drug CPT-11 and their anticancer activities were determined and compared with free drug. The cytotoxic activities are in the order of CTAB@MSNs > SDBS@MSNs > Triton@MSNs. The cationic CTAB@MSNs could have interacted with silanol groups on the silica surface and the drug for more focused actions to produce higher anticancer activities than the other surfactants and the free drug [87]. The anticancer drug, DOX, loaded onto MSNs via a physical absorption process, has been combined with cancer-targeting polypeptide 2,3-dimethyl anhydride (DTCPP) and a therapy peptide 2,3-dimethyl anhydride (DTPP). The pH-sensitive DOX@MSN-ss-DTPP&DTCPP NPs drug carriers exhibit the release behavior influenced by an acidic pH and glutathione (GSH). A high cytotoxicity towards HeLa cells is observed after 48 h treatment where the release of DOX and the therapy peptide stimulates apoptosis, targeting the cell nucleus and mitochondria [88]. A study on the controlled-release mechanism of the drug shows direct correlation of the decomposition of the silica nanostructure with the drug release from the porous structure. This diatom biosilica is biodegradable and cost-effective as compared to the synthetic counterparts, for theranostic applications, and the delivery of chemotherapeutics [89]. Curcumin (CUR) and 5-fluorouracil (5FU) loaded onto the MSNs with magnetic bacterial iron oxide nanowires, and further encapsulated in pH-sensitive hypromellose acetate succinate microspheres using droplet-based microfluidics, have exhibited synergistic effects on SW480 colon adenocarcinoma cells [90]. An advanced dual DDS (dDDS), based on R5 peptide-fused ferritin (R5FT) with biosilica deposition on the ferritin surface, has been developed to overcome the limitation of different physicochemical properties of the different drugs during combination drug therapy. DOX is loaded into the core inside (the ferritin cage), and the monomeric red fluorescent protein (mRFP) or paclitaxel (PTX) is retained by the biosilica matrix (Figure 6). The mRFP or PTX exhibits short-term release, while DOX shows a sustained release. The compartment-based and rate-controlled dDDS with the PTX-DOX combination attains a two-fold higher cytotoxicity than the single DOX [91].

Mesoporous silica (MCM-41, SBA-15, MCF-17, and MCF) synthesized by the hydrothermal method, having different pore sizes, surface areas, and structures, and functionalized with 3-mercapto propyl trimethoxy silane (MPTMS) followed by the oxidation of the thiol groups of the sulfonic group, has shown different loading capacities of tamoxifen citrate (TMXC) and release rates. A higher loading of TMXC is attained in the SO_3_H-functionalized SBA-15 and MCM-41. A fast release is however shown by the SO_3_H-functionalized MCF in acidic conditions and in the presence of NaCl [92]. The ~300 nm size of MCM-41 silica particles on a magnetic iron oxide template has been modified with NH_2_ or COOH groups, before or after the template removal, and then further modified with PEG-chains, for use as a DDS. The amount of TMX drug loaded in the carrier and its release characteristics depend on whether the modification step takes place before or after the template removal step. The TMX interacts more strongly with the silanol groups on the silicate surface than the surface-modified COOH groups, but sustained release is shown by the PEGylated formulation with COOH group modification after the template removal [93]. Mesocellular foam (MCF) with a spherical, continuous 3-D pore structure has been synthesized based on a Pluronic 123 triblock polymer (P123) surfactant and CTAB co-surfactant, for an oral drug delivery study. The MCF exhibits a higher telmisartan loading capacity and a faster release rate than the fibrous SBA-15 [94]. An antipsychotic drug, paliperidone-loaded mesoporous silica foam incorporated into polylactic acid and polylactic (co-glycolic acid), has resulted in enhanced drug solubility, but also prolonged release time [95]. The different performance of these functionalized silica-based drug carriers suggests the need to tune the carrier for a specific environment and to meet the intended purpose of the cargo to be delivered.

**Figure 7:**
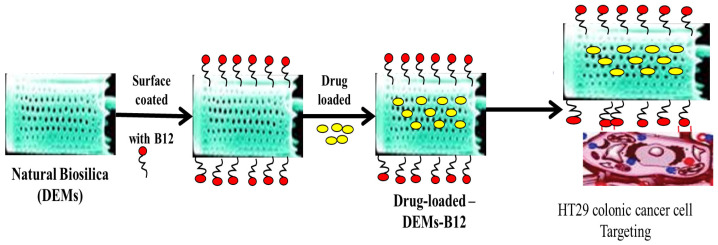
Natural biosilica “DEMs” coated with B_12_ and loaded with poorly water-soluble drugs. The loaded micro-shuttles target and interact with HT29 colon cancer cells before releasing the drug [99] (modified with permission, Copyright © 2018, Royal Society of Chemistry).

Diatom species produce potent bioactive compounds exhibiting anti-cancer, anti-biofilm, anti-inflammatory, and antibacterial activities [96]. Marine diatom *Thalassiosira weissflogii* frustules have been envisaged as a SMART DDS, where CUR is loaded at the capacity of almost 79%. Physiological conditions are found more suitable for faster CUR release, as compared to the acidic conditions. CUR adsorption into the void pores of biosilica is attributed to the surface charge of −3.05. The CUR-loaded biosilica shows cytotoxicity towards human renal adenocarcinoma cells (ACHN), but no toxicity against the human embryonic kidney (HEK293) cells, and with a broad spectrum of antibacterial activities against *Escherichia coli*, *Streptococcus pneumoniae*, *Staphylococcus aureus*, *Bacillus subtilis*, and *Aeromonas* spp. [97]. Biosilica extracted from *Chaetoceros* sp. diatoms with the surfaces modified with iron oxide NPs and functionalized with Trastuzumab antibody, has successfully exhibited fluorescence emission and could specifically target the SKBR3 (HER2-positive) breast cancer cells and not the normal cells. The *Chaetoceros* sp.-derived biosilica is eco-friendly and a promising biomaterial for biosensing chips and targeted drug delivery [98]. Marine microalgal-based diatomaceous earth microparticles (DEM) coated with vitamin B_12_ (cyanocobalamin) and loaded with cisplatin (CPT), 5-FU, and a tris-tetraethyl [2,2-bipyridine]-4,4-diamine-ruthenium(II) complex, are synthesized for targeted delivery to HT-29 colorectal cancer cells and MCF-7 breast cancer cells. CPT and 5-FU show rapid losses from the carrier, while the ruthenium complex is retained for up to 5 days in aqueous media, but readily released in a lipophilic environment as found in the cell membrane. The B_12_-modified DEM exhibits a three-fold higher adherence to HT-29 and MCF-7 cells, attributable to increased transcobalamin II receptor expression, and is suitable as a micro-shuttle interacting with the tumor site before unloading the drug (Figure 7) [99].

Diatomite biosilica has been developed as a nanocarrier to transport siRNA into the cancer cells. The microporous frustules are crushed, sonicated, and filtered up to a 450 nm size and surface-engineered with siRNA conjugation. The frustule carriers are proven as non-toxic materials for the transportation of siRNA into the tumor cells [100]. Iron oxide nanoparticles (IONP) have been loaded onto diatom frustules (IONP-DTM) and attracted to the tumor site of the in vivo animal model via an external magnetic field. Diatom sizes of 10 µm are used for the IONP and molecule loading, but the size may be large enough to cause blockage in the narrow capillaries of the lung, and the large pore size leads to cargo losses, suggesting the need to optimize the NP sizes. The broad range of diatom functionalities and the low toxicity and biodegradability of the IONP-DTM, suggest the great potential for its application in multimodality imaging and as a therapeutic vehicle in combination therapy [101]. CUR-loaded magnetically IONP-active frustules have exhibited reasonably higher cytotoxicity against the human cervical cancer (HeLa) cell line as compared to the pure CUR [102]. The DNPs nanovectors can be taken up by the cells and acclimatized to the lipid environment of the cell membrane, without damaging the cell morphology and viability [103]. The physicochemical and biological characteristics of the DNPs modified with 3-aminopropyltriethoxysilane (DNP-APT) have been improved using the dual-surface functionalization method by PEG layer and bioconjugation with cell-penetrating peptides (CPP). These are meant to enhance biocompatibility of the DNP-APT and its subsequent intracellular uptake by the cancer cells. The PEG-ylated and CPP-bioconjugated DNP-APT exhibit biocompatibility with erythrocytes, but a reduced cytotoxicity against the MCF-7 and MDA-MB-231 breast cancer cells. However, CPP conjugation enhances the DNP cellular uptake, and the dual biofunctionalization improves sorafenib drug-loading by 22% and the release profiles in aqueous system [104]. The genetically engineered *Thalassiosira pseudonana* diatom displays an IgG-binding domain of protein G on the surface of the biosilica to capture the cell-targeting antibodies. Only specific antibodies are attached to the drug-loaded biosilica NPs to target and selectively kill neuroblastoma and B-lymphoma cells. With similarly designed biosilica treatment, a regression in the tumor growth is also observed in the subcutaneous mouse xenograft model of neuroblastoma [105].

Living diatom cells suspended in buffer in darkness have very little silica dissolved from their cell walls, even if left for many weeks, suggesting the diatom resilience under a harsh environment and that there are intrinsic mechanisms stabilizing the diatom silica walls [106]. Diatom silica microparticles could improve permeation and sustained release following the oral delivery of drugs [107]. However, the biocompatibility and safety on human health upon treatment are of paramount importance, and many in vitro and in vivo tests are designed to test the biocompatibility of the material. The determination of cytotoxicity and mutagenicity during the in vitro analysis requires in vivo animal testing to improve injectable materials before further validation during clinical testing for later human use. This safety aspect is ever so pertinent in the application of siRNA-diatomite nanoconjugate for cancer treatment. The effects of siRNA should be disease site-targeted such that the normal cells will not be damaged. In vitro experiment has shown that diatomite NP concentration up to 300 ug/mL for 72 h shows very low toxicity, but the siRNA-diatomite nanoconjugate exhibits cytoplasmic localization and causes gene silencing in human epidermoid cancer cells (H1355) [100]. Biosilica accumulation has been detected in the kidney and the liver of the BLAB/c mice after 8 days, although no major distortion is observed in major organs [105], suggesting greater caution should be taken should there be the case of overdose and particle accumulation in tissues and organs in the body [4]. Apart from the use to induce cytotoxic and anticancer activities through drug delivery, biosilica can also be explored for synergistic applications with the drugs and biocompounds for reduced systemic toxicity during treatment [108]. For tissue engineering applications, biosilica from marine sponges has been shown as a promising biomaterial for bone graft due to the great potential to exploit its osteogenic properties. Biosilica has a positive impact on the viability of MC3T3-E1 osteoblast precursor cells, and stimulates Runx2 and BMP4 (Bone morphogenetic protein 4) expressions, the genes responsible for giving instructions to make proteins for the development and maintenance of teeth and bones [109].

## 6. Rational Design and Stimuli-Responsive MSNs

Biotemplated silica synthesis is the step forward for a more rationally designed silica-based material for DDS, tissue engineering, and biosensing applications [110]. Biotemplating makes use of nature-inspired techniques to develop new functional materials to overcome some of the limitations associated with current methods. In this case, diatom is a superior template for DDS, especially in the delivery of poorly water-soluble drugs, attributable to the highly ordered silica structure, species-specific architecture, and tunability for surface engineering. Drugs are physiosorbed or chemisorbed onto the surfaces and released via concentration gradient-driven diffusion. Pharmaceutical formulations, such as in the form of amalgams or compressed tablets, therefore influence disintegration or degradation for the controlled-release of the drug [111]. The use of nanocarriers in the treatment of human diseases, such as cancer, allows for site-specific targets, improved medication localization, with reduced drug dosage and systemic side effects [100]. To enhance the efficacy of treatment, the delivery of multiple drugs through nanocarriers has been proposed as a promising approach. The development of a nanocomplex for multiple drug delivery may require different types of switches to trigger the release of the right cargo, at the right time, and at the right site. A pH-sensitive MSN has been developed, decorated with pH-sensitive chitosan and lactobionic acid to target asialoglycoprotein receptors on hepatocellular carcinoma cells, for the co-delivery of sorafenib, a multi-tyrosine kinase inhibitor, and ursolic acid, which is to sensitize sorafenib. The designed nano-DDS has been suggested to enhance drug bioavailability and efficient targeting of tumor cells, with a pH-responsive function for sustained drug release [112].

Despite promising potential as a DDS, slow degradation and metabolism of silica-based drug delivery is the major bottleneck that must be overcome [113]. Uniform mono-dispersed 3D-dendritic MSN has been proposed to biodegrade rapidly in a simulated environment based on the biphase stratification strategy, and also be completely eliminated within 24 h [114]. However, the issue of rapid biodegradation must be addressed together with the slow-release mechanism such that the drug can be delivered in a correct dosage at the intended site. The common approaches in combining the biomacromolecules (BMs) with the NPs are through surface functionalization, chemical bonding, or electrostatic interaction between charged counterparts. The silica functionalization by condensation using alkoxysilanes should be performed prior to demoulding. The modification of the mesopores can be carried out either by condensation during template-based synthesis of the MSNs, or after surface modification and demoulding. To enhance degradation or slow-release characteristics, functional nanostructures can be prepared with sensitive bonds, nanopores, recognition elements, and charged surfaces capable of electrostatic interactions [115]. The grafting of the MSNs surfaces can be accomplished by direct condensation of the remaining Si–OH groups with silane functional reagents, usually the modified trialkoxysilanes with aliphatic chains carrying another functional group. For protein and peptide binding, carboxylate and amine can be used for amide conjugation and malimides for thiol-mediated binding [116]. Both the BMs and the nanocarriers should have strong electrostatic interactions from the differently charged functional groups. The nucleic acids (NAs) with permanent negative charges allow for strong reactions with the polymers and positively charged surfaces such as chitosan or polyethylenimine [115].

**Figure 8:**
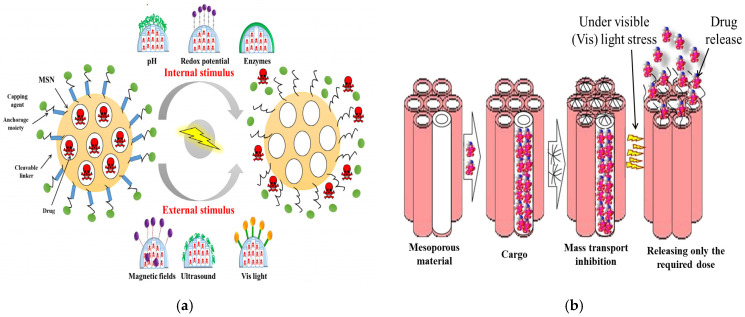
Stimuli-responsive drug delivery based on the MSNs: (**a**) The endogenous-stimuli response (pH, redox potential, and enzyme); and exogenous stimuli-response (magnetic fields (AMField), ultrasound (US), and visible light (Vis)) (adapted from [117] under Creative Commons Attribution (CC BY) license); (**b**) The loading and controlled drug release of the MSNs (adapted from [67] under Creative Commons Attribution (CC BY) license).

The nanoscale systems based on MSNs can be designed by blocking the pore openings using gatekeepers to prevent premature release of the cargo. Capping agents such as cyclodextrin, manifolds, polymers, and peptides/proteins could release the drug with the introduction of specific stimuli. There are two types of stimulus reactions—endogenous and exogenous stimulus responses. Stimulant-responsive characteristics can be achieved by fixing the caps that clog the pores via bonds that can be cut off when exposed to endogenous stimuli such as pH, redox potentials, or enzymes, which can be specific to the treated pathology; or via exogenous stimuli such as magnetic fields, ultrasound, or light, which can be applied by the clinicians as illustrated in Figure 8a and listed in Table 3. The MSNs that respond to internal stimuli do not require external devices to initiate cargo release. However, the precise drug dose control may be less than that achieved with external stimuli. Various aspects therefore must be considered such as the type of the mesoporous matrix, the types of drug, the target tissue environment and pathology, and of particular importance is the potential clinical application and how the drug is contained until it reaches the target area, before releasing the required dose (Figure 8b) [117]. The smart MSNs are capable of responding to the specific endogenous stimuli such as pH differences, a high GSH concentration, the overexpression of specific enzymes, or the presence of many small molecules. Generally, these intelligent DDSs have one or two components—the sensitive links and/or capping factor [117]. The responsive link is able to degrade, break, or undergo in harmony with the change initiated by the given stimulus. Capping agents such as organic or inorganic NPs, macromolecules, or polymers, block the inlets of the mesopores and impede early cargo departure, and are capable of decomposing under the influence of stimulation, allowing pores to be removed and the drug released [117].

### 6.1. Endogeneous Stimuli-Responsive Drug Release

#### 6.1.1. pH

pH has been widely explored to stimulate drug release as the human body has a wide pH range. The pH-sensitive nanocarriers are classified into polymeric nanocarriers (nanogels, polymeric drug conjugates, core-shell polymeric NPs, and micelles); liposomes; and inorganic NPs. Different types of cationic and anionic polymers are used to release pH-sensitive drugs such as poly(acrylic acid) (PAA), poly(aspartic acid) (PASP), poly(ethyl acrylic acid) (PEAA), methylglutarilate poly(glycidol), poly(methacrylic acid) (PMAA), and poly(l-histidine) [118]. A pH-response for drug delivery targeting the colon has been developed based on bifunctional succinylated ε-polylysine-coated (SPL) MSNs. The pH-responsive SPL serves as a nanogate to release the drug prednisolone, selectively, at the pH 5.5–7.4 of the colon pH condition, but not at pH 1.9 of the stomach or pH 5 of the small intestine. The SPL-coated NPs also deliver a cell membrane-impermeable dye, sulforhodamine B, intracellularly to the immune cells (RAW 264.7 macrophages) and the intestinal epithelial cancer cells (LS 174T and Caco-2 adenocarcinoma cells). These will be highly advantageous for the treatment of inflammatory bowel diseases or colorectal cancer [119].

#### 6.1.2. Redox

Redox release used to deliver anti-cancer drugs is based on internally present reducing agents and the linker such as the redox disulfide bonds. The synthesis of the disulfide-linked polyethylene glycol (PEG) is attached to the MSNs to release an oxidative-responsive drug. The PEG surface modification of the NPs confers significant biocompatibility [11]. The activity of the prepared MSNs has been estimated in a study on the in vitro release of Rhodamine-B (RhB) as a model drug, where GSH is added to the release medium in addition to the intracellular concentration. In the absence of GSH, the RhB release is insignificant, indicating the efficacy of the cap in preventing drug release. The intracellular GSH level is 2–10 mM, which is normally 100–1000 times higher than that found in humans and blood. However, the level of cytosolic GSH in some cancer cells is at least four times higher than in normal cells. The potential to design GSH-sensitive NPs can be developed based on the acute differences in GSH levels between tumor and normal cells. After cellular uptake, the cleavage of disulfide NPs with techniques sensitive to GSH can elevate intracellular drug delivery or genes, and this ultimately controls the intracellular fates of the delivered drugs and genes [120].

#### 6.1.3. Temperature

Temperature-responsive MSNs, based on poly(ethylene oxide)-b-poly(*N*-isopropylacrylamide) copolymeric micelles, have been developed as structure-guiding factors for the synthesis of functional MSNs loaded with model drug into the mesopore NPs. Using one-pot syntheses, the drug is directly combined into the hybrid material. The structure-directing factor in this process is the drug-loaded polymer micelles. The release of the drugs at 20 and 45 °C suggests a temperature-sensitive model of drug release [121]. The effects of pH-responsive DDS may need to consider the physiological state and temperature in the microenvironment. The in vitro pH-responsive behaviours of DOX-loaded hyaluronic acid modified with glycyrrhetinic acid and l-histidine at 37 °C suggest that the NPs are stable at pH 7.4, and after 24 h, release only 21.4% of DOX. At pH 6.8 of the extracellular tumoral environment, the cumulative DOX released is 29.8%, and at pH 5 of intralysosomal condition, 58.9% of DOX released and at a faster rate [122]. Due to inflammation, tumors, or infection, the mild temperature may increase by 4–5 °C. A pH-controlled release system should be designed to respond to the temperature by grafting a temperature-sensitive nano switch onto the surface of MSNs. The usually used heat-sensitive polymers are based on poly-*N*-isopropylacrylamide (PNIPAM) and its derivatives. These polymers, however, show a bottleneck that impedes drug release under the lower solution temperature [121].

#### 6.1.4. Chemical and Enzyme

Many chemicals and enzymes that are naturally present in the body or that are produced during disease states have also been examined for the potential to stimulate drug release from the MSNs. Glucose is a potential trigger for drug release, such as for the controlled-release of insulin and cyclic AMP, especially in managing diabetes [123]. Enzymes play key roles and support the pathology of many diseases, and have selectivity towards specific substrates, allowing well-defined bioreactions. Most enzymes catalyze reactions under moderate conditions (neutral pH, buffered aqueous poly-*N*-isopropylacrylamide solutions, and low temperature) where many traditional chemical reactions fail [124]. The unique properties of the enzyme include an isoelectric pH, substrate specificity, and a greater expression in certain organs and in semi-cellular organelles. During large changes in physiological conditions, such as inflammatory and disease conditions, intracellular proteases may play a crucial role, and the mineral proteins matrix may be specific to the cancer micro-environment. The concentration of elastase increases during inflammation, while phospholipase is excessively excreted in the case of pancreatic cancer, which can be of great use to deliver antibiotics. An oxidase-responsive DDS can be developed based on oxidoreductases. MSNs can be designed, using these different enzymes, by altering the bonds and blocking agents during surface functionalization [125]. The MSNs have been functionalized with a signaling reporter, the alizarin complexone (ALC) compound, where crystalline insulin is then introduced into the pores by benzene-1,4-dibronic acid (BP) mediated through an esterification reaction. This acts as a hypoglycemic agent as well as a pore blocker. Additionally, rosiglitazone maleate is also introduced into the pores to form multi-functional MSNs. If glucose is present, a competitive association occurs between the ALC and BP, which causes the pores to open and release the insulin, with real-time monitoring capabilities [126]. Another new chemical-responsive MSN uses thrombin, where the MSNs are loaded with an anticoagulant drug (acenocoumarol) and the pores are covered with LVPRGSGGLVPRGSGGLVPRGSK-pentanoic acid (P), a substrate of α-protein-hydrolyzed human thrombin. The MSN-based DDS is shown to be very sensitive to the presence of thrombin, where thrombin causes the hydrolysis of the blocking peptide to release the drug [127].

### 6.2. Exogeneous Stimuli-Responsive Drug Release

In comparison to endogenous stimuli, exogenous stimuli such as heat, magnetic fields, ultrasonic waves, and light, allow for a safer and more effective controlled release, as well as the ability to counteract the effects of internal individual variances. The physicochemical changes in the materials can be sped up for the temporal control of intended therapeutic benefits through exogenous physical stimulations such as in the case of photothermal and photodynamic therapies, thus preventing premature drug release and reducing the side effects [128].

#### 6.2.1. Magnetic Field

**Figure 9:**
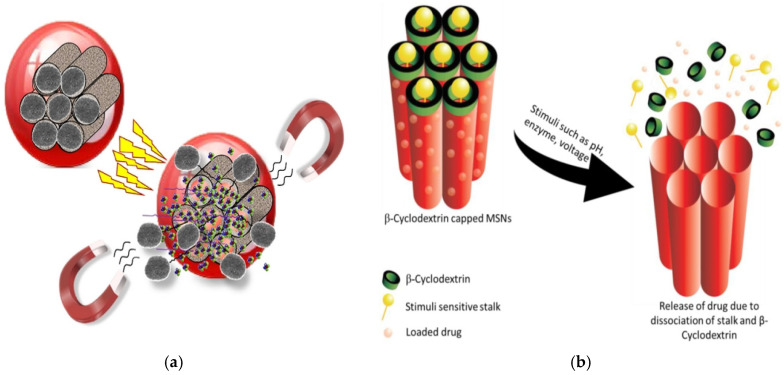
Stimuli-responsive drug delivery based on MSNs—(**a**) Release of drug from β-CD-capped-MSNs in response to endogenous stimuli (reproduced from [67] under Creative Commons Attribution (CC BY) license); (**b**) The drug is loaded into the mesopore ducts. The pore openings are then closed with nanocaps to block the early release of the cargo. At the target site, the stimulation removes the gatekeepers, allowing the trapped drug to be released (reproduced from [11] under Creative Commons Attribution (CC BY) license).

Iron oxide NP-encapsulated diatoms with small molecules could be magnetically delivered to the tumor site [101]. The advantages of using magnetic fields are the various manipulations and wide range of possibilities achievable with the MSNs through magnetic orientation under a permanent magnetic field or an increase in temperature when an alternate magnetic field (AM) is applied [129]. The magnetic NPs widely used for drug delivery that respond to the stimuli are the superparamagnetic-iron oxide NPs (SPIONs). These NPs are capable of converting magnetic energy into heat via Brownian fluctuations caused by rapid rotation of the magnetic nuclei, and Nell fluctuations caused by the rotation of the magnetic moments [130]. In magnetic resonance imaging and magnetic targeting, the MSNs are used to deliver the photosensitizer to the target site [131]. The incorporation of magnetic MSNs with stimuli-responsive components in a single nano-structural vesicle make it feasible to control the location, time, and amount of released drug as shown in Figure 9. Magnetic hyperthermia therapy is a cancer treatment that depends on heat created by the magnetic NPs under an alternating magnetic field to achieve less scar, with localized therapy and minimal side-effects [131].

Magnetic MSNs can control the localization of nano-transmitters through an external magnetic field. The alternating magnetic fields have been used to stimulate the controlled-release of fluorescein, where the ability of the DNA chains to hybridize inversely in response to the temperature changes is used as a gatekeeping strategy [132]. This temperature-responsive action can be controlled by changing the chain length, differences in G/C content, and grafting intensity on the material surfaces [133]. The pre-loaded fluorescein magnetic silica NPs are functionalized with 15 single oligonucleotide base pairs, carefully designed to exhibit a melting temperature with their integral strands in the hyperthermia range (45 °C). The system is coated with magnetic superoxide carrying the integral oligonucleotide sequence, avoiding the early release of fluorescein enclosed in the silica matrix. The application of an alternate external magnetic field causes dehybridization between the oligonucleotide filaments due to the increase in temperature, allowing the release of the fluorescein. Then, the fluorescein released could be stopped by simply removing the magnetic field as the hybridization between the integral strands occurs once the temperature drops below the fusion value, suggesting that the operation is intermittent at the nanoscale level [67].

#### 6.2.2. Ultrasound

Ultrasound (US) is an effective method to achieve spatiotemporal control of drug delivery at the target site and preventing damage to the healthy tissues. The US application is cost-effective, non-invasive, precludes the use of ionizing radiation, and facilitates the controlling of tissue penetration depth by adjusting the parameters such as the power, cycles, frequency, and exposure times [134]. A drug release from the MSNs could be triggered through the thermal effect, and via the specially designed MSNs with mechanophores or chemical bonds that split when induced by the US waves. The effect of high-intensity focused US (HIFU) has been used as a strategy to disassemble the amphiphilic diblock co-polymer (BCP) micelles in aqueous solution. A small amount of HIFU-labile 2-tetrahydropyranyl methacrylate (THPMA) co-monomer units introduced into the BCP, could be released with a HIFU irradiation without actually changing the solution temperature [135]. The polyethylenimine-coated US-responsive MSNs have shown the migration capability towards mammary tumors in vitro, and the release of DOX triggered only when the DDS is exposed to the US [136].

#### 6.2.3. Light

Light provides non-invasive and spatiotemporal control in the design of stimuli-responsive MSNs to carry out on-demand drug release upon illumination of a specific wavelength such as ultraviolet (UV), visible (Vis.), or near-infrared (NIR) regions. The advantages are low toxicity, ease of application, and accuracy for the desired location. However, the main drawback is the lower ability to penetrate into the tissue. The light irradiated-responsive MCM-41 MSNs modified by azobenzene derivatives could store small molecules and only release them upon light exposure. The β-cyclodextrin and/or pyrene-modified β-cyclodextrin rings bind to the trans-azobenzene cap of the nanopores, and dissociate from the stalks upon irradiation to release the cargo [137]. The most widely used light-responsive MSNs for drug release are based on UV as the UV can easily break the bonds, but may cause toxicity with low tissue penetrability [117]. The interest in the use of Vis. light is attributed to its higher penetrating capacity. A singlet-oxygen sensitive MSN-designed with porphyrin-caps grafted by ROS-cleavable linkages has been reported. The DDS releases the cargo upon exposure to the Vis. light from a common lamp. The singlet oxygen molecules produced by the Vis. light porphyrin-nanocaps, break the linker to release topotecan in a controlled-fashion manner inside osteosarcoma cancer cells, to achieve double antitumor effects that are based on the impact of the drug and the ROS [138].

## 7. Drug Loading, Release, and Cellular Uptake

While encapsulation or attachment of the drugs may not be a problem, chemical instability and unexpected leakage of the drugs have hindered rapid progress of the field. The use of MSNs for controlled DDSs could attain an optimal drug concentration over a sustained period, with enhanced treatment efficacy and reduced side effects [117]. MSNs have excellent biochemical and physico-chemical stability, biocompatibility, and endocytotic properties, to serve as effective carriers of anticancer drugs [3,13]. The stability of the MSNs could protect therapeutic agents such as proteins or RNAs from potential degradation, and the high loading capacity permits two or more drugs to be loaded in the same carriers, to deliver complex therapies to treat multidrug resistant tumors. The addition of contrasting agents for biomedical imaging is important real-time monitoring of the treatment. Careful control of cargo release from endogenous or exogenous responsive stimuli avoids early release of the therapeutic agent and reduces systemic toxicity [117]. Furthermore, the incomplete vascular structure and tumor lymphatic drainage/recovery system permits small nano-carriers and large particles to enlarge the endothelial barrier and aggregate in tumor tissue. The enhanced permeability and retention (EPR) effects are possible by passive targeting of the nanocarriers such as polymeric micelles, nano-capsules, liposomes, and NPs [3]. Active targeting is feasible via ligands on the surface of the nanocarriers such as mannose, folate, monoclonal antibodies, lactobionic acid, galactose derivatives, hyaluronic acid, transferrin, or arginine–glycine–aspartate, to attain a specific affinity for the receptors that are overexpressed on the surface of the target cells [3].

**Figure 10:**
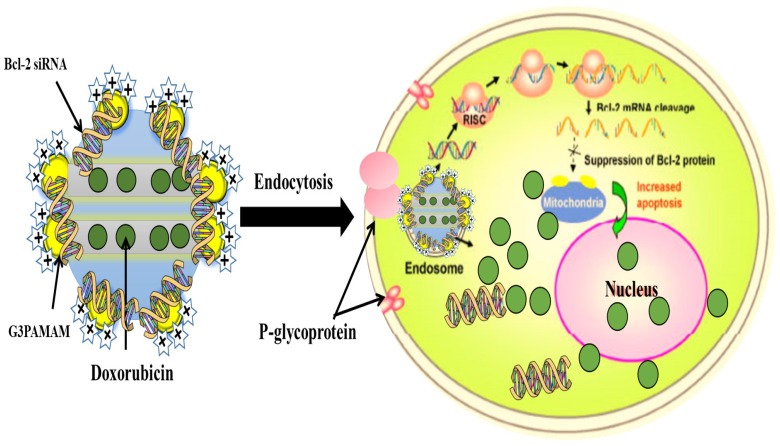
Co-delivery system based on MSNs to deliver DOX and Bcl-2-targeted siRNA simultaneously to A2780/AD human ovarian cancer cells for enhanced chemotherapeutic efficacy [140] (modified with permission, Copyright © 2009, John Wiley & Sons, Inc.).

The diffusion of the molecules from the pores can be tailored to suit the biological target by modifying the surface of the MSNs. The critical factor is the interaction between the functional groups on the pore surfaces and the drug molecule. Surface functionalization coming before or after drug loading could yield different results. Drug loading, followed by surface functionalization with amine groups, has been shown to sustain the drug release better than the systems that are first functionalized then followed by drug loading [11]. MSNs have a high drug loading capacity and adjustable release mechanisms due to their internal pores and large surface areas, which can be selectively designed to load hydrophobic or hydrophilic anticancer agents, and the size and shape can be controlled to increase the cellular uptake [139]. Different payloads can be introduced into the MSNs by loading on-site during manufacturing, or adsorption of loads into the mesopores in the MSNs via hydrogen or chemical bonding [12]. Gluconic acid-modified insulin has been covalently attached to the boronic acid groups present on the surface of the MSNs. The loadings of the cyclic adenosine monophosphate (AMP) and gluconic acid-functionalized insulin are reported to be 27 and 64 µmol/g, respectively. The glucose-responsive MSNs make it possible for the controlled-release of insulin and cyclic AMP [123]. As shown in Figure 10, the delivery of DOX and Bcl-2-siRNA simultaneously to an A2780/AD human ovarian cancer cells system, based on MSNs, could address the problem of multidrug resistance of the cancer cells [140]. The incorporation of an active folate-targeting ligand on 48 nm multifunctional MSNs (PEG-MSNPs48-CD-PEG-FA) with DOX-loading significantly inhibits tumor growth in mice. DOX is released by the intracellular acidic pH and GSH, and the PEG-NPs-loaded-DOX shows better in vivo therapeutic efficacy than the DOX alone and the non-targeting NPs [141]. These confirm the effectiveness of MSNs to tackle a multitude of challenges related to cancer treatment.

Hyaluronic acid (HA) has been linked to the MSNs to specifically target cancer cells, with a high potential for drug loading and a sustainable drug release. The amine groups of the MSN-NH_2_ could react with the carboxylic groups of HA with a 4-(4,6-dimethoxy-1,3,5-triazin-2-yl)-4-methyl-morpholinium chloride (DMT-MM) crosslinker. The HA-MSNs are absorbed at almost a three times higher rate than the free MSNs in squamous cell carcinoma 7 (SCC7) cells. To improve the anticancer effects of chemotherapy and photodynamic therapy (PDT), DOX and chlorine e6 (Ce6) are loaded in the HA-MSNs (DOX/Ce6/HA-MSNs) where the formulation demonstrates a higher cytotoxicity on the SCC7 than the free DOX, HA-MSNs-DOX, or Ce6 alone, suggesting significant therapeutic effects of chemo-PDT [142]. The HA-MSNs synthesized via a facile amidation reaction has been proposed as an efficient strategy to overcome the MSNs agglomeration problem in physiological fluids. A hydrophobic camptothecin drug encapsulated into the HA-MSNs exhibits selective targeting with enhanced cytotoxicity against HeLa cells, which is suggested to be due to the rapid uptake by the cancer cells through the CD44 receptor-mediated endocytosis mechanism. The low expression of CD44 in MCF-7 and L929 cells, however, has not resulted in selective targeting of the HA-MSNs [143]. Hyaluronidase-responsive MSNs have been developed as a DDS for cancer therapy. Desthiobiotin is grafted onto the MSN surface, followed by the generation of a streptavidin complex and functionalization with biotin-modified HA. DOX release in vitro from the HA-MSN-DOX system has been shown to be accelerated significantly with the presence of biotin and hyaluronidase, and induces apoptosis higher than the free DOX, where the uptake by the cancer cells is attributed to CD44-receptor-mediated endocytosis. The HA-MSN-DOX also inhibits tumors in vivo with low systemic toxicity [144]. The PDT-based MSNs with poly-(l-lysine) and HA coating is effective, even at a low fluency of PDT (14 J/cm^2^) and a low MSN concentration of 20 μg/mL against over-expressed CD44 receptors in HCT-116 colorectal cells. Excessive free HA, on the other hand, could have occupied the CD44 receptor on the cancer cells, which reduces the impact of the HA-MSN-PDT, suggesting the uptake by the cancer cells through endocytotic mechanism [145].

Active targeting of docetaxel (DTX)-loaded lactosaminated MSNs (Lac-MSNs), for asialoglycoprotein (ASGPR) receptors on hepatoma HepG2 and SMMC7721 cells, has been reported. The Lac-MSNs have a highly ordered hexagonal mesoporous structure with a high surface area of 1012 m^2^/g, an average pore size of 3.7 nm, and are cytotoxic and endocytosed by the ASGPR-positive hepatoma cells but exhibit non-selective endocytosis with the ASGPR-negative NIH3T3 cells. The clathrin-mediated pathway is suggested to predominate the energy-intensive cellular uptake and internalization process [146]. Copper-impregnated MSNs (Cu-MSNs) with 3-amino-1,2,4-triazole (AT) as a catalase inhibitor, has been exploited to achieve ROS-mediated apoptosis to kill cancer cells. The presence of AT increases the hydrogen peroxide level and free radicals in colon carcinoma HT-29 cells treated with Cu-MSN-AT. The synergistic effect of catalase inhibition by the Cu-MSN-AT and the ROS-triggered apoptosis represents targeted therapy with reduced toxic effects from the non-specific ROS as observed in conventional chemotherapy [147]. The complete inhibition of tumor growth in human pancreatic cancer xenografts of different mice species has been achieved upon treatment at the minimum dose of 0.5 mg camptothecin (CPT)-loaded MSNs/mouse, where biocompatibility, renal clearance, and a high efficacy of drug delivery is observed. Significant improvement is also reported with folic acid-modified MSNs [148]. The folic acid loaded on the surface of MSNs is possible through an amide linkage between the carboxyl group of folic acid and the amine group of APTES. The folate-modified MSNs/CPT result in a higher cytotoxicity against SK-BR-3 cells than MCF-7 cells, due to the higher expression level of folate receptors on the SK-BR-3 cells. Without folate modification, the MSNs/CPT delivery is not specific and attains a similar cytotoxicity against the two cancer cells. The folate-mediated active targeting of folate receptor-positive tumor cells by the MSNs therefore have great potentials in treating tumorigenesis [13,149,150]. The DDS based on MSNs, chitosan, and poly(lactic-co-glycolic acid) could reduce toxicity, increase bioavailability and the half-life of drugs and proteins, improve the solubility of hydrophobic drugs, and allow controlled and targeted delivery of anticancer agents and therapeutic genes at the diseased sites [1,3]. The encapsulation of MSNs loaded with poorly water-soluble felodipine has been reported using chitosan and acacia (ACA) by a layer-by-layer self-assembly process. The felodipine release rate decreases with increasing layers of chitosan/ACA coated on the MSNs. The drug release rate can therefore be controlled through multilayer coating, and the different behaviour under various pH conditions reflects the different states of the multilayer [151].

The sustained-release within 24 h of the nanohybrid diatom silica (DE) and GO, functionalized with APTES, where 70.8% of IMC released, is achieved with GO-DE fabrication at pH 7.4, and 31.1% with GO-DE at pH 3.5, as compared to 87.7% APTES-DE, proves the feasibility of diatom-based silica as a smart DDS based on pH-responsive stimuli [83]. The IONP-loaded diatom frustules (IONP-DTM) for magnetic delivery to the tumor site in vivo, have also been proven successful, although the large 10 µm sizes may require smart plugs to prevent drug leakage before stimulation is introduced. The NP sizes also need to be optimized during processing and fabrication to prevent blockage of the narrow capillaries of the tissues or organs in the body, and to attain better pharmacokinetics [101]. The applicability of diatom frustules from *Aulacosiera* sp. functionalized by dopamine-capped-IONP for a magnetically driven DDS has been shown. The slow release of IMC as an entrapped drug fits the zero-order kinetics model where the drug is suggested to be released in a controlled fashion with a uniform amount in each time unit, typical of the reservoir transdermal delivery systems [152]. Precise functionalization of silanated (APTES, octadecyl trimethoxysilane (OTMS), and 3-cyanopropyl triethoxysilane (CPTES)) hollow MSNs (HSMN), with amine, carboxyl, cyano, or methyl groups onto the HMSN, could control the 5-FU drug loading capacity. With amine grafting, for example, the highest loading capacity of 28.9% is attained as compared to the normal HMSNs (18.34%). This is due to similar hydrophilic characteristics of the functionalized MSNs but of contrasting charges through electrostatic interactions between 5-FU (negatively charged) and HMSNs (positively charged). The higher drug loading at pH 7.4 and 8.5 is attributed to 5-FU being deprotonated in alkaline conditions [153]. The pore swelling agents such as tri-isopropylbenzene (TIPB), alkanes/ethanol, trio-octylamine (TOA), *N*,*N*-dimethylhexadecylamine (DMHA), and decane can help to increase the pore sizes [11]. Two approaches—drug loading first, surface functionalization later (DL-SF), and surface functionalization first, drug loading later (SF-DL), based on the APTES silylation of MSNs, have produced different results. The DL-SF MSNs achieve a 75% encapsulation efficiency of DOX, with the in situ formation of an APTES external layer restricting the leakage of DOX under physiological conditions. The DOX-loaded DL-SF MSNs show a negatively charged surface but becoming positively charged under an acidic tumor microenvironment. The SF-DL MSNs, in contrast, result in the reduction of DOX loading due to the APTES layer, which decreases the inner permeability of DOX [154]. Co-condensation and post-synthesis functionalization methods of the MCM-41 further suggest that the different methods used result in varying distributions of functional groups on the MCM-41 surface, which interact with the drug molecule to different extents and therefore influence the drug release properties [155]. Eventually, this regulation of preparation conditions allows for both temporal and spatial control of the drug release [69].

## 8. Advanced Applications

### 8.1. Gene Therapy

Naked nucleic acids (NAs) show minimal penetration into cell membranes and a carrier is required for gene transfection. Gene therapy for cancer treatment especially lacks specific delivery systems to transport the therapeutic genes to target cancer cells [156]. There are two important gene delivery systems—the viral and non-viral systems. There are some safety concerns with regards to the viral systems due to the possibility of genetic recombination, immunogenicity, and lack of specificity. Because of this, the non-viral systems that include recombinant proteins, cationic compounds, polymers, and inorganic NPs, have been extensively explored [3]. MSNs are particularly attractive for gene delivery due to their unique properties that could increase the efficiency for cell adsorption and transfection [3]. The silica-based vectors are safe, cost-effective, and stable with a modifiable surface and structure [156]. More importantly, the bioconjugated MSNs can protect the DNA from cleavage by the nucleases, thus ensuring the integrity of the genes delivered [157]. PEI is widely used in gene delivery systems due to the amino proton that improves gene delivery, with facilitation for endosomal escape. The PEI-coated cationic MSNs show strong binding affinities for DNA and siRNA, with a high transfection efficiency of up to 70% in the cells. PEI can also be coupled to other particles prior to attachment to the MSNs to control gene release [158]. Liposomes have been used to enhance the cellular uptake of the NAs. However, both polycations and liposomes are facing some limitations attributable to their molecular weight, gene escape, and physico-chemical instability. MSNs, on the other hand, have shown optimal intracellular delivery characteristics of the NAs, and the MSNs in combination with polycations and other curative medicines can be harnessed for personalized treatment related to cancer and other diseases [115]. Antisense oligonucleotide-loaded MSNs are found effective in inhibiting the growth and survival of the HeLa cells [159]. The DNA or siRNA attached to the PEI-modified MSNs have been applied for gene therapy. The green fluorescent protein (GFP)-containing siRNA is used as a delivery model where the MSNs coated with 10 kDa PEI exhibit the best transfection efficiency in transporting the GFP-siRNA into the tumor cells [158]. The co-delivery of miRNA-204-5p (tumor inhibitor miRNA) with oxaliplatin (OXL, anticancer drug) in PEI/HA-loaded MSNs (Oxmi-HSMN) exhibits synergistic anticancer effects on colon cancer. PEI is attached to the MSNs through electrostatic interactions, resulting in increased miRNA loading. The covalently-linked HA to the MSNs surface permits selective targeting of CD44 receptors on the colon cancer cells [160].

Unmodified MSNs have negative charges due to the silanol groups on the surface that reduce the binding to the negatively charged DNA. The modification to increase the net positive charges, which include cationic polymer functionalization and co-delivery with metal cations, enhances gene loading by enhancing the electrostatic interactions between modified-MSNs and the NAs [3]. Polymers such as poly-l-lysine (PLL) could carry large DNA, penetrate cell membranes with a low immunity, and be degraded by enzymes for controlled-release behavior. The PLL-MSNs, as an enzyme-stimulated system, can therefore control simultaneous drug and gene release. The positively charged modified-MSNs establish high electrostatic interactions with the negatively charged cell membrane, leading to increased cellular uptake. It is important to regulate the amount of cation polymer in order to achieve a balance between transfection activity and toxicity of the modified-MSNs system for gene delivery [3]. MCM-41, with small pores (2–3 nm), have the genes or plasmids being limited by the small pore size, with the genes being mainly adsorbed on the outer surface, instead of being loaded into the pores. These have resulted in gene leakage. Additionally, the genes on the outside of the MSNs could be degraded by the lysosomes or nucleases. The NPs, with large pore sizes, help to attain internal gene storage and protection [161]. The synthesis of MSNs with large pores can be achieved by controlling the temperature or pore swelling agents such as 1,3,5-trimethybenzene (TMB). The MSNs with large pores (~23 nm) show the ability to protect the plasmids from nuclease degradation with a higher transfection efficiency as compared to the MSNs with small pores (2.1 nm) [161]. The MSNs with large pores of 9 nm, synthesized using ethanol as a co-solvent, a fluorocarbon–hydrocarbon surfactant as a template, and modified with a hydrophobic octadecyl group, exhibit a high loading capacity for surviving siRNA delivery to prevent colon cancer cell proliferation [162]. Both the net surface charges and the pore sizes are therefore important factors to be optimized for the MSNs to serve as effective carriers for the delivery of genes and plasmids.

### 8.2. Biomedical Imaging and Theranostic Applications

**Figure 11:**
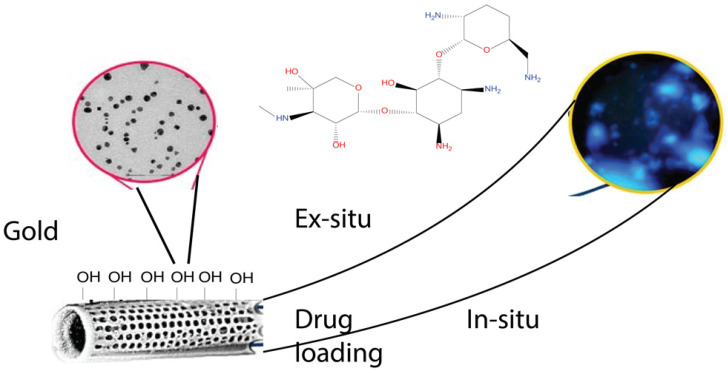
Image of stained biosilica exhibiting high porosity in the structure [164] (reproduced with permission, Copyright © 2019, American Chemical Society).

The ability to carry multiple compounds within a single carrier, and their controllable stability and size, make MSNs an excellent platform for biomedical imaging and diagnostic and therapeutic applications. Fluorophores with a poor stability and solubility, particularly for near-infrared (NIR) imaging, can be combined into the MSNs with other compounds to enhance their photophysical and photochemical features [11]. Diatom frustules functionalized with special luminescent and light emitting molecules can serve both as drug carriers and to evaluate the release characteristics of the cargo from the luminescence property. In vivo modification of *Thalassiosira weissflogii* diatoms biosilica with red-emitting dyes containing phenyl, benzothiadiazolyl, and thienyl groups, have been successfully accomplished. The highly luminescent biosilica is obtained from the metabolic reaction of the dyes within the diatom shells, followed by a, acidic–oxidative method of isolation of the stained biosilica [163]. Silica-based nanostructures from *Thalassiosira weissflogii* frustules have been incorporated with iridium (Ir-1), a phosphorescent organometallic complex, for diverse applications such as photonics, diagnostics, and imaging. The biofactory strategy through the in vivo biomineralization of orthosilicic acid is proposed as an economical and sustainable route of production for silica nanomaterials to be doped with heavy metals, transition metals, or rare-earth metals [164]. The method of preparation of the functionalized MSNs has been demonstrated to be important in determining the rate of the drug released. As illustrated in Figure 11, diatom biosilica of *Aulacoseria* sp. decorated with gold NPs and loaded with gentamicin (GMC) exhibits a fluorescent intensity based on the amount of GMC loading. The in vitro drug release characteristic in simulated body fluid is traced using UV–Vis spectrophotometry. The ex situ method of preparation is shown to result in a lower GMC loading but a faster release rate, while the in situ method exhibits a longer and slower release rate. This finding could pave the way for a more rational design and control of the decorated structure and optical properties of the diatom biosilica for drug delivery [165].

Diatom frustule ordered porous structures have a broad range of applications for the fabrication of nanostructured templates and nanoimprint lithography, such as for medical implants where nanosize precision is of paramount importance [166]. The hierarchical design of a diatom frustule-inspired nanostructure utilized in metamaterial absorber fabrication has produced nano-resonators with a broadened, strong absorption band of the spectrum [167]. These unique nanostructures of the MSNs can be of great assistance in PDT. The photodynamic activity against tumor-associated macrophages can be reportedly achieved through covalent bioconjugation of a photosensitizer C-phycocyanin (CPC) to biosilica under a laser irradiation of 620 nm. The incorporation to biosilica has significantly improved the non-toxic CPC for PDT application [168]. Diatoms such as *Chaetoceros* sp. have optical properties specifically in the form of a fluorescence emission, which will be of great use in bioimaging. Biosilica from *Chaetoceros* sp., modified with IONPs and functionalized with trastuzumab antibody to differentiate between normal and breast cancer cells, could be magnetically driven for the detection and delivery of the drug to the targeted site [98]. Photoluminescent (PL)-active biosilica from diatom *Pinnularia* sp. has been developed as a biosensor, through modification with a single chain variable fragment from anti-2,4,6-trinitrotoluene (TNT) monoclonal antibody, to detect TNT in aqueous solutions. The biosensor emits visible blue PL, but upon binding of the TNT to anti-TNT fragment-functionalized biosilica, the PL emission is partially quenched so that the dose–response curve can be drawn to estimate the level of TNT. The detection is also selective for TNT and trinitrobenzene [169]. Biosilica from *Thalassiosira weissflogii* and surface coated with polydopamine (PDA) films, fluorescent labeling and silver NPs are designed for multifunctionalities in bionanomedicine, with antimicrobial and photonic properties [170]. The mesoporous silica microcapsule-supported silver (Ag) NPs show the dispersion of the AgNPs within the mesoporous silica shell structures, which prevent the clustering of the AgNPs, as normally observed in the colloidal system. The slow release of the Ag^+^ ions through diffusion from the pores could sustain a high level of antibacterial activity over the period of two months [171].

**Figure 12:**
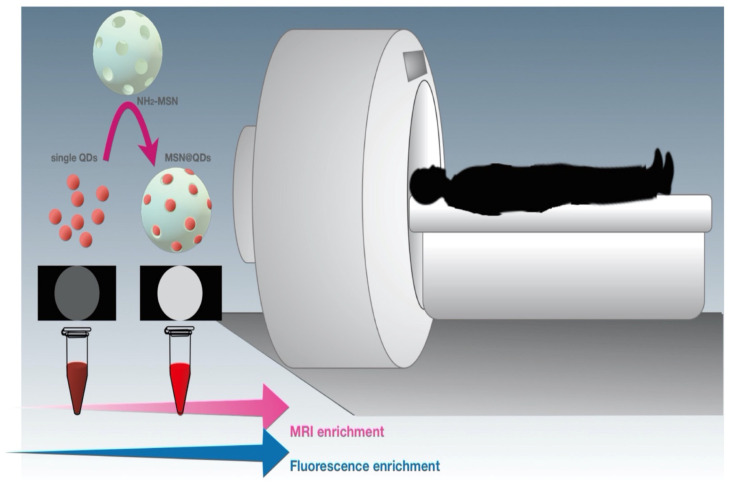
MSNs as a dual imaging probe [172] (reproduced with permission, Copyright © 2012, American Chemical Society).

MSNs can be used for optical imaging, ultrasound imaging, magnetic resonance imaging (MRI), positron emission tomography (PET), computed tomography (CT), and multimodal imaging. Optical imaging allows the focused area to be identified by the incident light, stimulated usually in the visible or NIR region, which is emitted at a lower energy [11]. A combination of the dyes also with the MSNs could give adequate stability and protection from the environment. Squaraine-loaded MSNs, for example, are covered with graphene oxide thin sheets where the pigments have significant photo-responsive characteristics in the NIR region. The study with HeLa cell lines suggests that the developed hybrid platform protects the dye and prevents it from escaping from the system [172]. MRI is a potent non-invasive in vivo imaging that gives a high-resolution 3D anatomical and functional image of the focused area (Figure 12). The MSNs-based contrast agents lead to a higher sensitivity for MRI, due to the large specific surface areas of the MSNs that could carry higher payloads. The MSNs functionalized with target ligands could efficiently bind to the damaged tissues and be observed under a relevant imaging system [173]. In CT application, an NIR-developed fluorescence/CT dual-modal imaging probe allows the MSNs-loaded Au nanoclusters (NCs) to target oral cancer cell lines SCC-25, CAL-27, and ACC-2. Compared with the normal AuNCs, the MSN@AuNCs show improved NIR/CT fluorescence efficiency with a high sensitivity for disease diagnosis and treatment [174].

The imaging agents may not work in some cases due to a low penetration depth, a poor sensitivity, and a low resolution. The applicability and accuracy at the specific sites can be improved by combining ultrasound with MRI, or optical imaging with MRI, CT, or PET [173], such that the limitation in one technique can be complemented by the advantages in another technique. MSNs, with active surface moieties possessing imaging and drug delivery properties, can be directed to abnormal tissues for therapeutic and diagnostic purposes. The drug-loaded MSNs with the MRI contrasting agent 19F, labeled with fluorescent dyes, and linked with folic acid, is designed for enhanced delivery, uptake, and monitoring in the cancer cells. The cellular uptake by folate-expressing cancer cells could be monitored by the 19F MRI and fluorescence microscopy [175]. SiNPs incorporated with quantum dots (QDs), forming a core envelope of CdSe@ZnS for bioimaging purposes in the form of Si@QDs@SiNPs, have exhibited superior fluorescence as compared to the QDs alone in HeLa cells. An in vivo assay for which the mice are injected with Si@QDs@SiNPs mixed with HeLa cells, shows improved fluorescence, confirming the effectiveness of the design for bioimaging and cell tracking with a high sensitivity [176]. Cyanine 7-doped-SiNPs for mapping of the lymph node using NIR imaging, and also for diagnosing potential diffusion and draining nodes, further suggests the importance of surface chemistry architecture to improve the efficiency of the mapping [177].

## 9. Issues and Challenges

Multidrug resistance (MDR) prevents effectiveness of the drug prescribed for treatment, and in cases such as cancer, is the major obstacle in chemotherapy. The MDR in tissues is complex, involving multiple dynamic mechanisms. There are generally two types of MDR: pump, and non-pump resistance. The pump resistance is attributed to the expression of drug-flow pumps, such as multidrug resistance protein (MRP1) and P-glycoprotein (P-gp), which flush out the drug to reduce the intracellular concentration of the drug [3]. The non-pump resistance, in the case of cancer, indicates the activation of the cell anti-apoptotic protein (Bcl-2) defense pathway, which decreases the drug sensitivity. Both resistance mechanisms, however, can interact mutually [3]. The enhanced cellular uptake and nuclear aggregation of DOX-loaded-MSNs in MCF-7/ADR cancer cells may have resulted from the overriding of the drug flow mechanism and/or down-regulation of P-gp by the MSNs, resulting in the cytotoxicity eight times higher than the DOX alone. Functionalized MSNs not only increase cell proliferation suppression in the ADR cells with DOX treatment, but are also capable of delivering multiple agents, such as anti-tumor and MDR reversal molecules [178]. Due to the nanoscale and unique properties of the MSNs, aided with a rational design process, MDR can be overcome through an enhanced cellular uptake, increased intracellular aggregation, and an elevated drug efficacy [179].

The combination of small molecule and macromolecule drugs could address MDR, but the major problem is to load them at the same time in a carrier and have the drug released individually. Conventional combination therapy in cancer treatment, for instance, does not meet the desired outcome due to these different drugs exhibiting varying pharmacokinetics. The use of a nanocarrier as a DDS in combination therapy offers great potential to entrap different drugs simultaneously to unify the pharmacokinetics of each drug, and to achieve site-specific co-delivery strategies [58,59]. Biodegradable nanocapsules based on an organosilica core-shell structure, have been designed to attain a dual stimuli-responsive DDS. The silica shell coated externally onto the macromolecule drug will be disrupted by a high GSH concentration within the tumor cells, allowing for the encapsulated drugs to be released. The small molecule drug coating the silica surface will be released based on the pH-responsive imine bonds in the acidic lysosomal condition. The designed multidrug carrier does not work in the normal cells as the responses needed to trigger the drug release are found only in the tumor microenvironment [59]. MSNs decorated with G2-polyamidoamine (PAMAM) simultaneously load DOX and Bcl-2 siRNA to address multidrug-resistant cancer cells. The treatment with MSN-Bcl-2 siRNA-DOX-G2-PAMAM confirms that Bcl-2 siRNA delivery silences the Bcl-2 mRNA expression and strongly suppresses the resistance, resulting in enhanced DOX anticancer activity [3].

The MSNs could carry different types of therapeutic and diagnostic agents simultaneously. There are several factors that can determine the biodistribution, stability, and pharmacokinetics of the MSNs such as a high drug-loading capacity, the ability to adjust loading/release mechanisms, and the chemical flexibility that allows for volume adjustment, charging, and surface functionalization [5]. The nano-platform offers diagnostic advantages through the targeted delivery of imaging agents to improve signal detection. However, PDT as non-invasive treatment requires photosensitizer (PS) drugs and an external light source. These hamper the rapid application of the PDT in clinical setting as PS drugs may lack tissue specificity and have poor water-solubility. Functionalized MSNs, in combination with PS drugs, could overcome these limitations [180]. Generally, MSNs used for the targeted delivery of signal/biomarker sensors could improve the detection of typically weak tumor biomechanics [5]. The development of an ultrasensitive MSN sensor can make use of the advancement in antibody–antigen interactions. The integration of gold NPs with diatom frustules for the ultrasensitive detection of interleukin 8 in blood plasma has been fabricated based on the surface-enhanced Raman scattering immunoassay. The enhanced localized surface plasmon resonance of the gold nanostructure is attributed to the large surface area, mesoporous, and ordered structure of the biosilica. The 5,5′-dithiobis(2-nitrobenzoic acid)-labeled immune-gold NPs act as the sandwich between the antibodies on the biosilica with interleukin 8 antigens, resulting in a high sensitivity and specificity of immune biomarker detection [181].

Many new drugs have a low solubility in water, leading to poor absorption. To enhance water-solubility and improve oral bioavailability, the MSN-based DDS provides effective solutions, such as the use of mesoporous silica SBA-16 whose morphology and synthesis time can be controlled through the addition of cetyltrimethylammonium bromide, and the pore size controlled by manipulating the heating temperature to deliver poorly water-soluble IMC [182]. The incorporation of poorly water-soluble clotrimazole into mesoporous silica structure is made possible using supercritical CO_2_ as solvent [183]. The etching time of the MSNs with a specific solution such as NaBH_4_ could be used as a strategy to modulate the pore size especially to cater for water-insoluble drug loading [184]. The increase in pore size between 3.7 and 16 nm of the 3D face-centred cubic mesoporous silica is shown to increase the dissolution rate of poorly soluble celecoxib [185]. To plug the different pore sizes, the design based on smart pH-responsive plug-gate nanovalves using peptide-functionalized MSNs should be considered to reduce drug leakage and for a more targeted delivery of the drug. The outer MSN shell is modified with K_8_ peptide(octa-lysine sequence), and the negatively charged surface is prepared by reacting the K_8_-peptide with citraconic anhydride, and the cationic K_8_ peptide is formed by the introduction of an Arg-Gly-Asp sequence to the K_8_-peptide-citraconic anhydride surface. At pH 5, representing the endo/lysosomal conditions, the nanovalves opening is induced to release DOX, while a lower DOX is released at a higher pH of 6.5 and 7.4 [186].

While the utilization of MSNs for targeted drug delivery and theranostic application has been shown to be promising, much work is needed to establish the dosage–toxicity relationship of the crystalline and amorphous micro-sized bulk silica and nano-sized silica, their interactions with the biological system, and the long-term impacts, before implementation in a clinical setting and especially for cancer treatment. In vitro studies of nanosilica have shown cellular uptake with size- and dose-dependant toxicity, elevated ROS levels, and enhancement of pro-inflammatory responses. Some in vivo studies demonstrate the possibility of reversible lung inflammation, with the formation of granuloma and focal emphysema, but with the absence of lung fibrotic development [187]. The exposure of the monodisperse, amorphous spherical silica NPs (14–16 nm sizes) has caused cytotoxic effects on human endothelial EAHY926 cells, with necrotic cell death within hours of exposure. Both particle surface area and shape are suggested to be important determining factors of the observed toxic effects [188,189]. A comparison with standardized materials is needed to establish the correlation between the physico-chemical characteristics of the different forms of nanosilica to the experimental data on toxicity [187]. To improve the economics of biosilica production from diatoms, the concept of the integrated biorefinery of algal cultivation should be considered [190], especially in meeting the agenda of global sustainable development goals and making disease diagnosis and treatment more accessible and affordable to the general masses.

## 10. Conclusions

The ability of the DDS to transport therapeutic molecules and be released in a controlled manner at the targeted site, without any loss in their activities and structures, is of paramount importance. MSNs and diatom biosilica have shown great advantages for diverse applications and to address the pharmacological needs in targeted therapy, especially with the latest development in cancer therapeutics, immunotherapy, and gene therapy. These are largely attributable to their unique, ordered porous structure, high intrinsic surface areas and pore sizes, high loading capacity, tunable release capability, biocompatibility, thermal and mechanical strength for stability, and the flexibility for functionalization and modification with different ligands and stimulus-responsive modifiers. The silica-based vectors have attracted attention in gene therapy for their safety and cost-effectiveness, modifiable surface and structure, stability, and the large porosity that can be designed to carry many genes, protect the genes from nucleases, and also increase cell transfection efficiency. The MSNs-based multifunctional systems show great potential to carry multiple drugs for combination therapy to overcome multidrug resistance prevalent in cancer treatment, or to deliver dyes or photosensitizer drugs that confer optical/photonic properties for the real-time monitoring and detection of the delivery to the target sites. However, there is currently a lack of reliable preclinical studies in vivo, methods for the NPs removal, and the extent to which the different shape, pore size and symmetry, contact with the biosystem, and chemical composition, would affect their effectiveness and toxicity during applications.

## Figures and Tables

**Table 3:** Stimuli-responsive drug release of MSNs and the mechanism involved.

Stimuli	Principle
pH	Depends on the pH of the tumor and inflammatory tissue, more acidic than that of the normal tissues and blood.
Redox	Based on the difference of redox concentrations of the normal tissues and tumors.
Temperature	Depends on the variation of the ambient temperature, the vessel expands and increases the cargo release.
Antibody	The pores covered with polyclonal antibody specific for the drug.
Enzyme	Regular expression profile of specific enzymes in disease conditions.
Light	Non-interference feature, spatio-temporal remote control.
Magnetic	Temperature-dependant, able to generate thermal energy.
Ultrasound	Sensitive polymer changing its water resistance, form coil-like gate opening, and cargo released after UV irradiation
